# Production of Single-Chain Fv Antibodies Specific for GA-Pyridine, an Advanced Glycation End-Product (AGE), with Reduced Inter-Domain Motion

**DOI:** 10.3390/molecules22101695

**Published:** 2017-10-10

**Authors:** Natsuki Fukuda, Kentaro Noi, Lidong Weng, Yoshihiro Kobashigawa, Hiromi Miyazaki, Yukari Wakeyama, Michiyo Takaki, Yusuke Nakahara, Yuka Tatsuno, Makiyo Uchida-Kamekura, Yoshiaki Suwa, Takashi Sato, Naoki Ichikawa-Tomikawa, Motoyoshi Nomizu, Yukio Fujiwara, Fumina Ohsaka, Takashi Saito, Katsumi Maenaka, Hiroyuki Kumeta, Shoko Shinya, Chojiro Kojima, Teru Ogura, Hiroshi Morioka

**Affiliations:** 1 Department of Analytical and Biophysical Chemistry, Graduate School of Pharmaceutical Sciences, Kumamoto University, 5-1 Oe-honmachi, Chuo-ku, Kumamoto 862-0973, Japan; nfukuda@seimeibunseki.org (N.F.); 173y1026@st.kumamoto-u.ac.jp (L.W.); kob@kumamoto-u.ac.jp (Y.K.); 530mihiro530@gmail.com (H.M.); ay1m2.vh.ucs@gmail.com (Y.W.); spz49kb9@gmail.com (M.T.); nakahara-yu@kaketsuken.or.jp (Y.N.); p1035_o83_ume@yahoo.co.jp (Y.T.); makiyo@ya2.so-net.ne.jp (M.U.-K.); ysuwa0115@gmail.com (Y.S.); tsato@kumamoto-u.ac.jp (T.S.); 2 Department of Molecular Cell Biology, Institute of Molecular Embryology and Genetics, Kumamoto University, 2-2-1 Honjo, Chuo-ku, Kumamoto 860-0811, Japan; noi@abc.me.es.osaka-u.ac.jp (K.N.) ogura@gpo.kumamoto-u.ac.jp (T.O.); 3 CREST, JST, 4-1-8, Honcho, Kawaguchi, Saitama 332-0012, Japan; 4 Graduate School of Environmental Earth Science, Hokkaido University, Kita-10 Nishi-5, Kita-ku, Sapporo 060-0810, Japan; naoichi@fmu.ac.jp (N.I.-T.); nomizu@toyaku.ac.jp (M.N.); 5 Department of Cell Pathology, Graduate School of Medical Sciences, Kumamoto University, 1-1-1 Honjo, Chuo-ku, Kumamoto 860-8556, Japan; fuji-y@kumamoto-u.ac.jp; 6 Graduate School of Pharmaceutical Sciences, Hokkaido University, Kita-12 Nishi-6, Kita-ku, Sapporo 060-0812, Japan; fohsaka@eis.hokudai.ac.jp (F.O.); saito-t@hokuyakudai.ac.jp (T.S.); maenaka@pharm.hokudai.ac.jp (K.M.); 7 Global Station of Soft Matter, Global Institution for Collaborative Research and Education, Hokkaido University, Kita-15 Nishi-8, Kita-ku, Sapporo 060-0815, Japan; kumeta@pharm.hokudai.ac.jp; 8 Laboratory of Molecular Biophysics, Institute for Protein Research, Osaka University, 3-2 Yamadaoka, Suita, Osaka 565-0871, Japan; shinya-s@protein.osaka-u.ac.jp (S.S.); kojima-chojiro-xk@ynu.ac.jp (C.K.); 9 Division of Materials Science and Chemical Engineering, Graduate School of Engineering, Yokohama National University, 79-5 Tokiwadai, Hodogaya-ku, Yokohama 240-8501, Japan

**Keywords:** GA-pyridine, single-chain variable fragment, phage display, isothermal titration calorimetry, differential scanning calorimetry, small-angle X-ray scattering, high-speed atomic force microscopy, NMR analysis, inter-domain motion

## Abstract

Due to their lower production cost compared with monoclonal antibodies, single-chain variable fragments (scFvs) have potential for use in several applications, such as for diagnosis and treatment of a range of diseases, and as sensor elements. However, the usefulness of scFvs is limited by inhomogeneity through the formation of dimers, trimers, and larger oligomers. The scFv protein is assumed to be in equilibrium between the closed and open states formed by assembly or disassembly of VH and VL domains. Therefore, the production of an scFv with equilibrium biased to the closed state would be critical to overcome the problem in inhomogeneity of scFv for industrial or therapeutic applications. In this study, we obtained scFv clones stable against GA-pyridine, an advanced glycation end-product (AGE), by using a combination of a phage display system and random mutagenesis. Executing the bio-panning at 37 °C markedly improved the stability of scFvs. We further evaluated the radius of gyration by small-angle X-ray scattering (SAXS), obtained compact clones, and also visualized open–close dynamics of these scFvs by high-speed atomic force microscopy (HS-AFM), revealing that one of the compact clones was biased to the closed state. Finally, nuclear magnetic resonance (NMR) analysis revealed that peak intensity and line width became homogeneous, supporting that dynamic features and/or formation of oligomers was improved in the thus-obtained clone. These findings should contribute to the future industrial and therapeutic use of scFvs.

## 1. Introduction

Currently, monoclonal antibodies are widely used for the diagnosis and treatment of various diseases, and as sensor elements [1,2,3,4]. Monoclonal antibodies require cultivation of mammalian cells for preparation, resulting in a high production cost. Single-chain Fv fragment (scFv) antibodies may offer a promising approach for overcoming this problem. These antibody derivatives are recombinant proteins consisting of a heavy-chain variable region (VH) and light-chain variable region (VL) connected by a short flexible polypeptide linker, and can be expressed by *Escherichia coli* (*E. coli*) and easily engineered by genetic techniques [5,6,7,8]. For rational design of high-quality scFvs, it is essential to clarify the interaction between antibodies and antigens in detail. To date, however, the use of scFvs has been limited due to their inhomogeneity, which is caused by their tendency to form dimers, trimers, tetramers, and larger oligomers [9,10,11,12,13].

**Figure 1:**
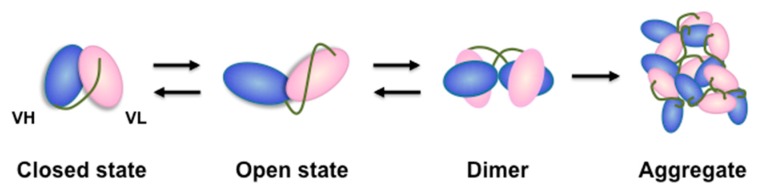
Schematic model of the aggregation process of scFv.

The scFv protein is assumed to be in equilibrium between a closed state, in which the VH and VL domains are assembled by inter-domain interactions, and an open state, in which two domains are disassembled (Figure 1). The open state causes inter-chain VH-VL interactions, which results in the formation of oligomers. The formation of oligomers, in turn, increases the apparent affinity for antigens through the avidity effect, resulting in a difference in activity between production lots. Further formation of huge oligomers is assumed to result in precipitation. For these reasons, the production of an scFv whose dynamics is biased to the closed state will be indispensable to the future industrial and therapeutic use of scFvs. 

**Figure 2:**
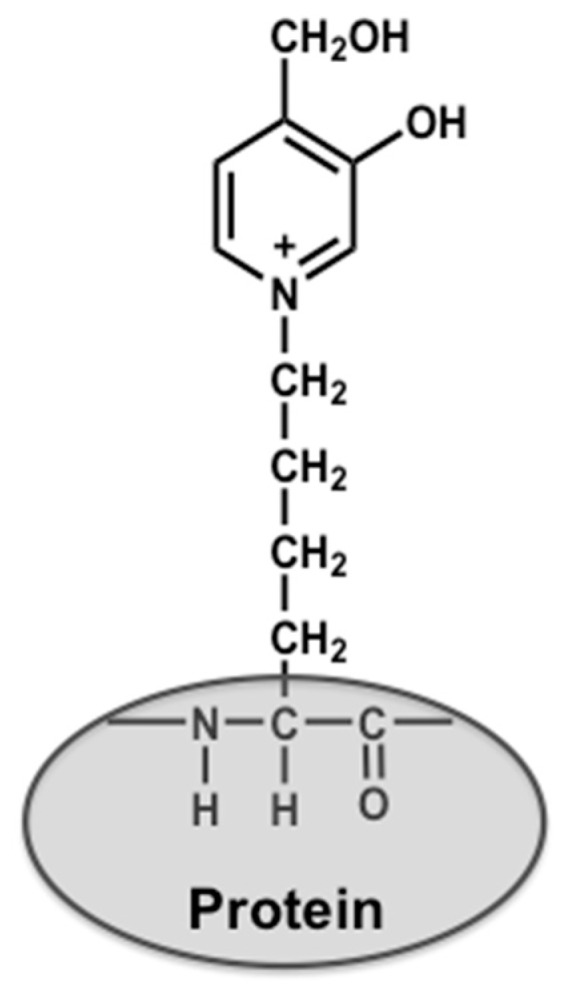
Chemical structure of GA-pyridine.

Increasing the interactions at the VH-VL interface is critical for shifting the scFv structure to the closed state to inhibit the formation of oligomers. The introduction of disulfide bonds into the VH-VL interface is one of the ways to maintain these interactions in cases where the structural information is already available [14,15,16,17]. Another method is to use a phage display system, which can be used without structural information. In the phage display system, scFv proteins are fused to the coat protein of the phage and displayed on the surface [18]. After several rounds of affinity selection from the phage library by means of bio-panning, clones exhibiting the desired properties are condensed. By changing the conditions of the bio-panning, it becomes possible to improve the biophysical properties of scFvs, e.g., stability, solubility, affinity, and yield [19,20]. However, the construction of a screening system for obtaining dynamically biased clones would be a highly difficult and complex undertaking. The stability of scFvs could be affected by inter-domain interactions, as well as by the intrinsic stability of the respective domains. Accordingly, we selected stable clones by executing the bio-panning at 37 °C for the initial screening, and evaluated the radius of gyration (denoted as *R*_g_) by small angle X-ray scattering (SAXS) to select a compact clone in the second screening step. We applied this strategy to an scFv against 3-hydroxy-4-hydroxymethyl-1-(5-amino-5-carboxypentyl) pyridinium cation (GA-pyridine), which is an advanced glycation end-product (AGE) that is generated by the Maillard reaction and occurs between the aldehyde residues of reducing sugars and the ε-amino group of the Lys residues of proteins (Figure 2) [21]. 

We obtained several compact clones whose *R*_g_ values were similar to the typical scFv in the closed state in the protein data bank (PDB IDs of 1n4x, 1p4i, 1x9q, 2kh2, 3juy, 4h0h, 4kv5, 4p48, 4x4x, 5i4f, 5jyl, 5jym, 5npi, and so on). We also performed HS-AFM to directly evaluate the open–close dynamics of scFvs, and confirmed that the finally obtained clone (73MuH19) exhibited reduced open–close dynamics as compared to the parental scFv, and addition of GA-pyridine further reduced the motion. Finally, NMR analysis was performed and the peak intensity and line width was revealed to be homogeneous, supporting that dynamic features and/or formation of oligomers was improved in the thus-obtained clone. Our results demonstrate that the combination of a phage display system plus SAXS and high-speed atomic force microscopy (HS-AFM) analyses for obtaining a clone that exhibits reduced open–close dynamics is useful for the production of an scFv sample for multidimensional NMR analysis, and our findings should, thus, contribute to the future industrial and therapeutic use of scFvs.

## 2. Results

### 2.1. Preparation of Anti-GA-Pyridine scFvs by Phage Display

**Figure 3:**
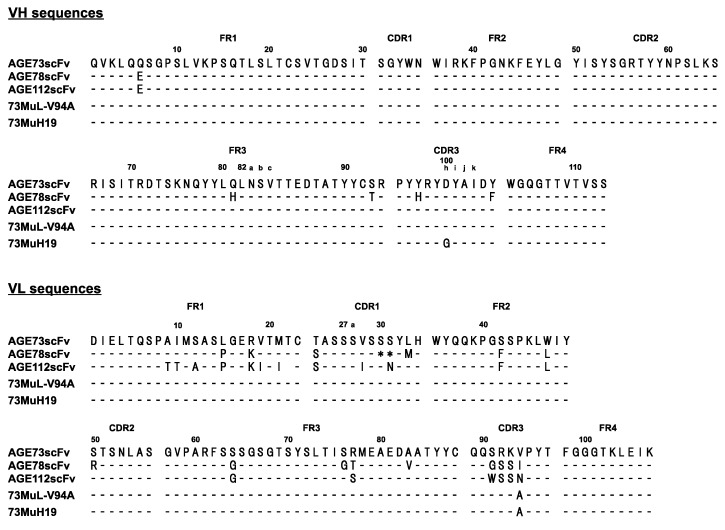
Amino acid sequences of the VH and VL of scFv clones selected by phage display. AGE73scFv, AGE78scFv, and AGE112scFv were obtained from the GA-KLH immunized mouse antibody library, which exhibited a positive response for GA-BSA in phage ELISA. 73MuL-V94A from the second library, and 73MuH19 from the third library were selected. Amino acid sequence differences are indicated; those that are conserved between the proteins are denoted by dashes.

We performed bio-panning using a glycolaldehyde (GA)-modified bovine serum albumin (BSA)-coated plate to obtain an anti-GA-pyridine scFv by means of a phage display system. The mouse antibody library immunized with GA-modified keyhole limpet hemocyanin (KLH) was displayed on the phage and used for bio-panning. Phage enzyme-linked immunosorbent assay (ELISA) showed that the reactivity of AGE73scFv to GA-BSA was the highest among the isolated clones (Figure S1). It should be noted that the amino acid sequence of the VH of AGE73scFv was highly conserved among the obtained clones (more than 96% in identity) (Figure 3).

**Figure 4:**
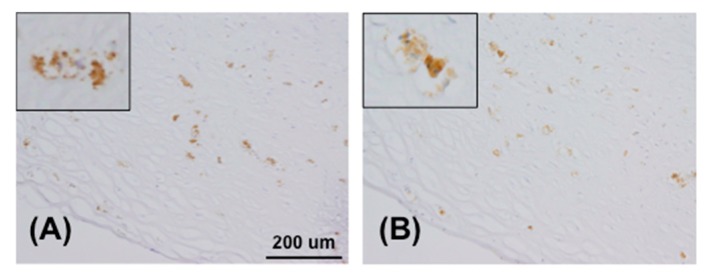
Immunohistochemical staining of human atherosclerotic lesion. Positive reactions were seen for the accumulation of GA-pyridine (brown). The sections were treated with anti-GA-pyridine monoclonal antibody, 2A2 (**A**) and AGE73scFv (**B**), respectively.

This result suggested that VH might contribute to the specificity for GA-pyridine. To confirm the activity of the AGE73scFv clone for GA-pyridine, we expressed AGE73scFv in *E. coli* and purified the resulting protein. The immunoreactivity of AGE73scFv to AGE-modified BSAs was analyzed by competitive ELISA. As shown in Figure S2, AGE73scFv exhibited a significant level of competition with GA-BSA, but competed only modestly with glyceraldehyde-modified BSA (GCA-BSA) or glucose-derived AGE-modified BSA (Glc-BSA), and only slightly with methylglyoxal-modified BSA (MGO-BSA), or glyoxal-modified BSA (GO-BSA). In previous reports, the accumulation of GA-pyridine in human atherosclerotic lesions was detected by immunohistochemical staining with the anti-GA-pyridine monoclonal antibody clone 2A2 (Cosmo-Bio) [21,22]. The results showed that AGE73scFv exhibited a reactivity to human atherosclerotic lesions similar to that of 2A2 (Figure 4A,B).

**Figure 5:**
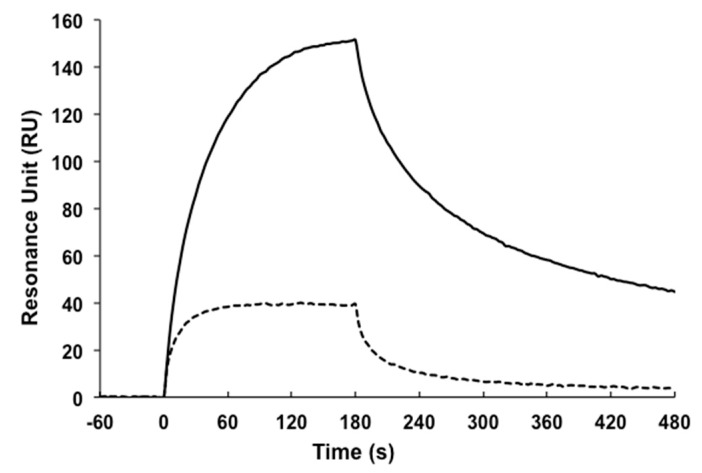
SPR analysis of the binding affinity of AGE73scFv. The binding affinity of AGE73scFv for the peptide containing GA-pyridine was measured at 25 °C (solid line) and 37 °C (dashed line).

In addition, we performed surface plasmon resonance (SPR) analysis of the interaction between AGE73scFv and the GA-pyridine-containing peptide immobilized on the sensor chip at 25 °C and 37 °C (Figure 5). AGE73scFv exhibited significant affinity for the peptide containing GA-pyridine at 25 °C, but only weak affinity for this peptide at 37 °C.

### 2.2. Selection of scFv with High Stability by Phage Display

To improve the stability, we constructed a second library by introducing random mutations into the VL domain of AGE73scFv. Error-prone PCR was performed under the condition that a one-to-three amino acid mutation was introduced into the VL region. To obtain stable scFv clones, the binding step of bio-panning was performed at 37 °C. After the fourth round of bio-panning, we analyzed the sequences of 48 clones, and 23 of these were found to be mutants. Phage ELISA revealed the high reactivity to GA-BSA of three mutant clones—73MuL-S30P, 73MuL-S56P, and 73MuL-V94A—respectively. Therefore, these three scFv clones were expressed in *E. coli* and prepared for further evaluation. Gel filtration chromatography, which was the final purification process, revealed that the retention time of the 73MuL-V94A mutant scFv was significantly delayed as compared to those of the other AGE73scFv mutant clones, suggesting that 73MuL-V94A possesses a more compact shape (Figure S3 and Table S1). We, therefore, focused on 73MuL-V94A and performed isothermal titration calorimetry (ITC) and differential scanning calorimetry (DSC) to further examine its affinity and stability. The 73MuL-V94A mutant exhibited about 2.5-fold higher affinity for *N*^α^-(carbobenzyloxy)-GA-pyridine (CBZ-GA-pyridine) than AGE73scFv at 25 °C, and about 4.5-fold higher affinity at 37 °C (Table 1 and Figure S4). However, the *T*_m_ value was similar between AGE73scFv and 73MuL-V94A (Table 1 and Figure S5). 

Based on the 73MuL-V94A mutant clone, we constructed a third library. A random mutation was introduced into the VH region of 73MuL-V94A, and another VH library was constructed. After the fourth round of the bio-panning, we analyzed the sequences of 28 clones from the VH library. There were seven mutated clones in the VH library. The 73MuH19 (H-D100hG) clone was selected in terms of frequency (Table S2), and characterized by ITC and DSC after purification in similar ways. The affinities for CBZ-GA-pyridine were similar between 73MuL-V94A and 73MuH19 (Table 1 and Figure S4). The melting temperature (*T*_m_) value of 73MuH19 was 65.0 °C, which was higher than the *T*_m_ of 62.7 °C for 73MuL-V94A (Table 1 and Figure S5). Therefore, it was considered that 73MuH19 might increase the inter-domain interactions. 

### 2.3. Evaluation of Molecular Size by SAXS

**Figure 6:**
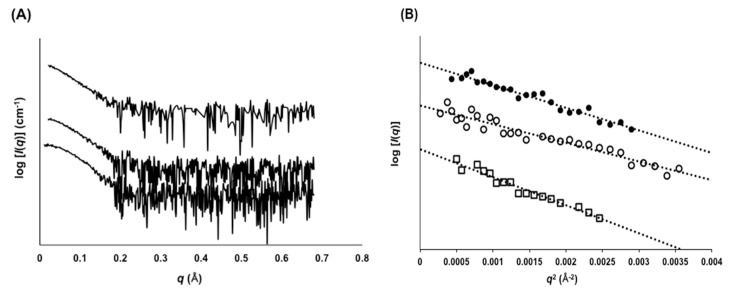
SAXS analysis of mutant scFvs. (**A**) Experimental SAXS curves of AGE73scFv, 73MuL-V94A, and 73MuH19 are shown from top to bottom; (**B**) The Guinier region and linear regression (dotted line) for *R*_g_ evaluation. The plots represent AGE73scFv (white squares), 73MuL-V94A (black circles), and 73MuH19 (white circles), respectively.

To clarify the details of the dynamic features of scFv, the *R*_g_ values were measured by SAXS (Figure 6A,B). The *R*_g_ value of 73MuH19 was 20.5 Å, while those of AGE73scFv and 73MuL-V94A were 25.1 Å and 22.5 Å (Table 1). The *R*_g_ value of anti-IL-1β scFv in the closed state was calculated to be 19.0 Å from the solution structure (PDB ID of 2kh2 [11]) by using CRYSOL software [23]. The *R*_g_ value of 73MuH19 was the smallest among the clones, but was slightly larger than the value calculated for anti-IL-1β scFv. It could be assumed that the equilibrium of 73MuH19 was biased to the closed state, or that all the molecules were homogeneously in the partially open state. 

### 2.4. Direct Evaluation of Open–Close Dynamics by HS-AFM

**Figure 7:**
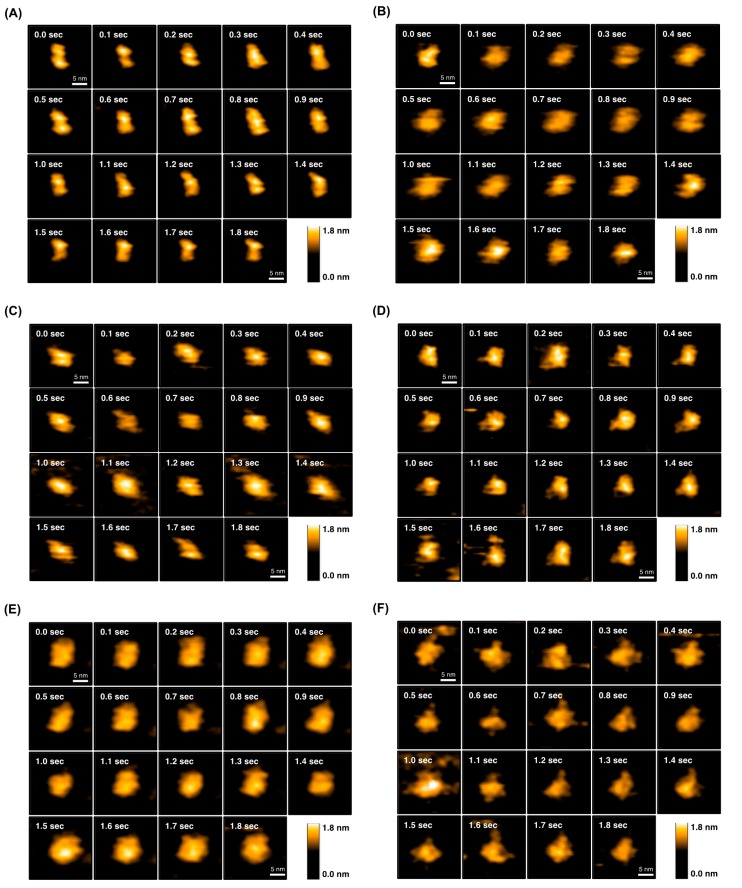
HS-AFM images of AGE73scFv, 73MuL-V94A, and 73MuH19. HS-AFM images of AGE73scFv, 73MuL-V94A, and 73MuH19 are shown in the absence (**A**,**C**,**E**) and presence (**B**,**D**,**F**) of CBZ-GA-pyridine, respectively.

**Figure 8:**
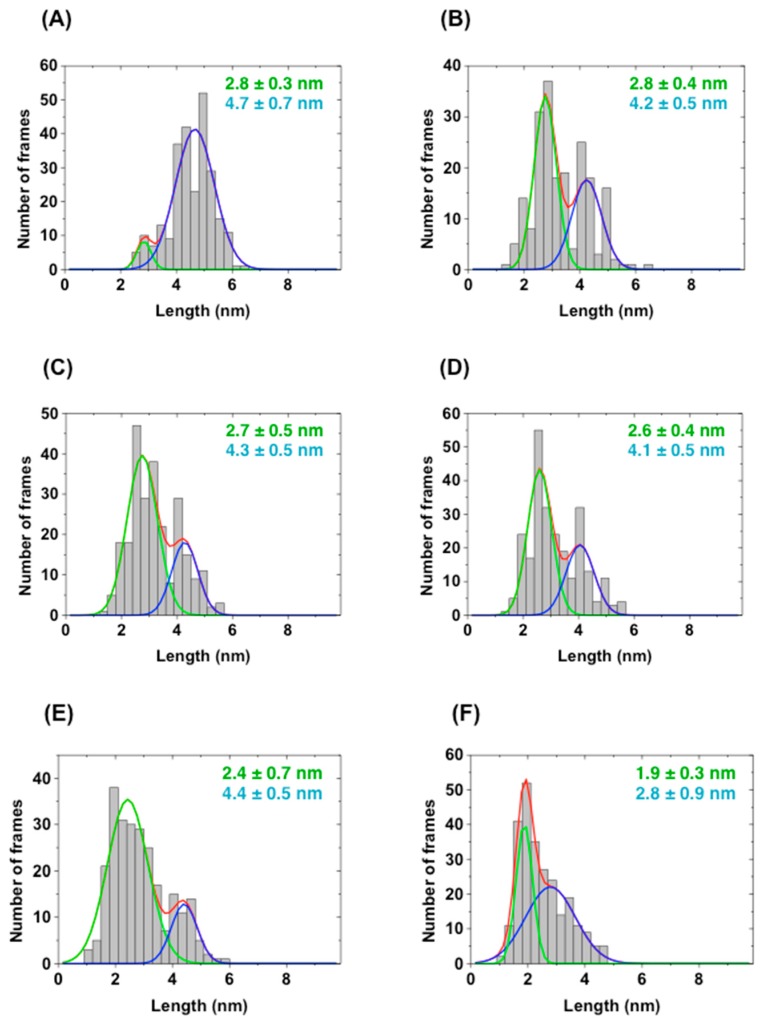
Histograms of the distances between the VH and VL domain. Histograms of the distances between the VH and VL domain were constructed from AFM images of AGE73scFv in the absence (**A**) and presence (**B**) of CBZ-GA-pyridine; 73MuL-V94A in the absence (**C**) and presence (**D**) of CBZ-GA-pyridine; and 73MuH19 in the absence (**E**) and presence (**F**) of CBZ-GA-pyridine. The distances between VH and VL domain were calculated by Origin software. The fitted distribution line for all scFv molecules (red), molecules with shorter distances (green), and molecules with longer distances (blue) are shown.

To further elucidate the open–close equilibrium of scFv, we performed HS-AFM analysis to directly observe the time-dependent conformational changes of AGE73scFv (Figure 7A,B), 73MuL-V94A (Figure 7C,D), and 73MuH19 (Figure 7E,F). HS-AFM is a powerful tool to visualize the dynamics of proteins on a millisecond time scale, and allows for the discrimination between open and closed states of scFv (Figure S6). The scFv clones were immobilized on a mica plate, and AFM images were taken every 100 msec. As a result, we observed that the molecular shape of AGE73scFv (Figure 7A) was clearly extended in comparison with that of 73MuL-V94A (Figure 7C) and 73MuH19 (Figure 7E). We constructed histograms of the distance between the VH and VL domains estimated from the AFM images of five scFv molecules for five seconds. In the histograms, most of the AGE73scFv existed at a length between 4 and 5 nm (Figure 8A), while 73MuH19 was mainly present at a length between 2 and 3 nm (Figure 8E). Based on the mathematical deconvolution of the histogram shown in Figure 8, more than 90% of AGE73scFv was in the open-state (peak at 4.7 nm), while about 80% of 73MuH19 was in the closed-state (peak at 2.4 nm) (Table S3). Following the addition of CBZ-GA-pyridine to AGE73scFv, the number of extended molecules was significantly reduced from about 90% (peak at 4.7 nm) to about 40% (peak at 4.2 nm) (Figure 7B and Figure 8B, Table S3). These data suggested that AGE73scFv was mainly in the open state without antigen, and this dynamic was suppressed by the addition of antigen, resulting in an equilibrium shifted to the closed state (peak at 2.8 nm). Similarly, the open state of 73MuH19, which was a minor component (about 20% with the peak at 4.4 nm in Figure 8E and Table S3), changed to a closed state (peak at both 1.9 and 2.8 nm) (Figure 7F and Figure 8F, Table S3). These results indicated that 73MuH19 is able to exist mainly in the closed state despite the absence of antigen.

### 2.5. NMR Measurements of 73MuH19

**Figure 9:**
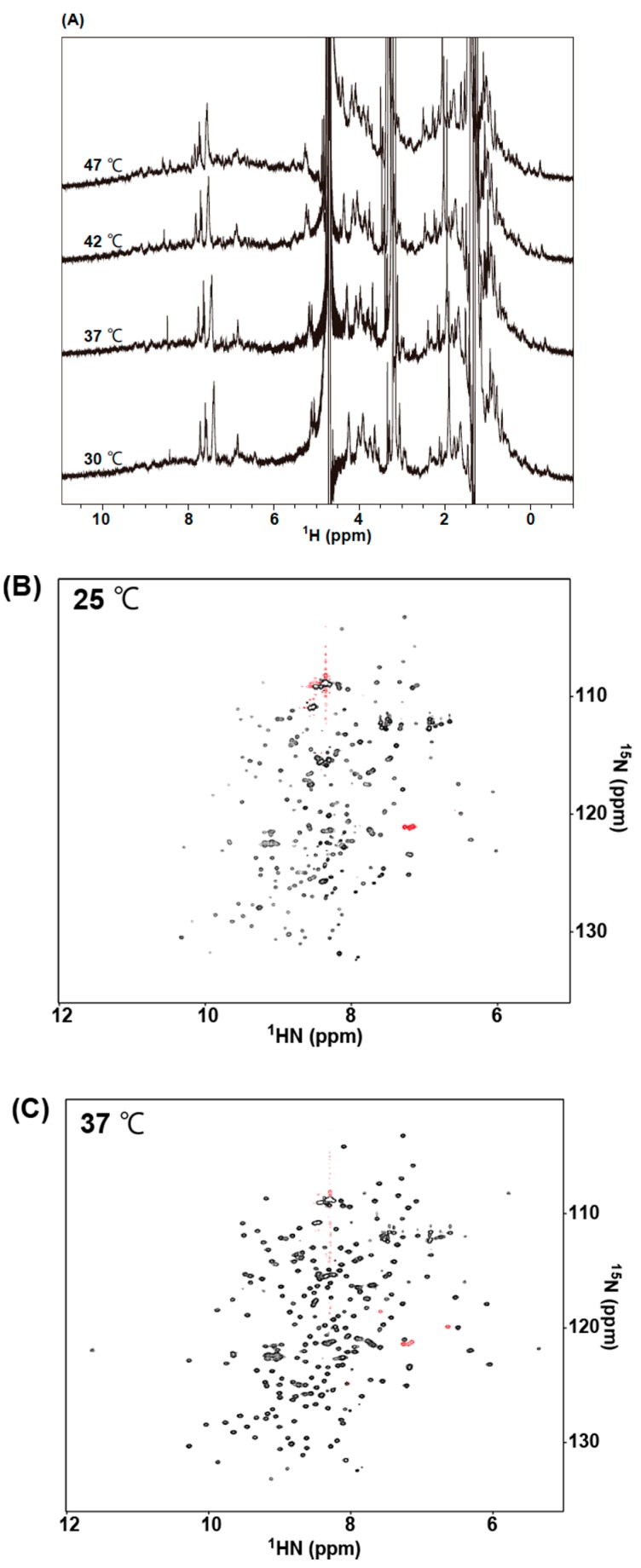
NMR spectra of 73MuH19. Proton 1D NMR spectra of 73MuH19 in the presence of CBZ-GA-pyridine at various temperatures ranging from 30 °C to 47 °C are shown in panel (**A**). ^1^H-^15^N HSQC spectra of 73MuH19 in the presence of CBZ-GA-pyridine at 25 °C and 37 °C are shown in panels (**B**) and (**C**), respectively.

Based on the results of various biophysical evaluations, we measured NMR spectra of 73MuH19 in the GA-pyridine bound state, and could successfully concentrate 73MuH19 up to 300 µM. Incidentally, the antigen free AGE73 could be concentrated, at most, up to 70 µM, and the antigen-free 73MuL-V94A, at most, up to 145 µM due to precipitation by the formation of large oligomers. Measurements of the 1D NMR spectra of 73MuH19 in the GA-pyridine bound state were made at several temperatures ranging from 30 °C to 47 °C. Under these conditions, the methyl signals around 0 ppm were not perturbed by increasing the temperature, revealing that the NMR sample of 73MuH19 in the CBZ-GA-pyridine bound state was stable up to 47 °C (Figure 9A). In the next experiment, the ^13^C/^15^N-enriched 73MuH19 was prepared, and the ^1^H-^15^N HSQC spectra were measured at both 25 °C and 37 °C (Figure 9B,C). The spectral quality was revealed to be better at 37 °C than at 25 °C (Figure 9B,C). The 73MuH19 exhibited well-dispersed spectra at both temperatures. Moreover, the peak intensity and line width was revealed to be similar in the whole region, indicating the reduced open–close dynamics and/or formation of the oligomers. The ^1^H-^15^N HSQC spectra of both the antigen free AGE73scFv and the antigen free 73MuL-V94A exhibited inhomogeneity in the peak intensity and line width, exhibiting appreciable open–close dynamics and/or formation of the oligomers (Figure S7).

## 3. Discussion

Here, we successfully obtained a stable clone, 73MuH19, as a third-generation scFv by using the combination of a phage display system and error-prone PCR. In previous studies, several strict conditions during bio-panning led to the production of scFv clones with high stability [19,20]. In the present study, we performed the bio-panning at 37 °C. ITC and DSC analyses suggested that this heating method could affect the stability in the third generation, and the affinity, rather than the stability, in the second generation. We considered that the affinity to GA-pyridine of 73MuH19 might reach a plateau, so that the high-temperature stress could significantly affect the stability. If bio-panning were performed at temperatures higher than 37 °C, more compact clones might be obtained. It should be noted that the filamentous M13 phage sustains the ability to infect up to 60 °C, and a higher temperature dramatically decreases the reinfection rate to *E. coli* [19].

Moreover, we evaluated the open–close dynamics of scFv clones by SAXS and HS-AFM. We succeeded in visualizing the open–close dynamics between the VH and VL domains by using HS-AFM, and found that the equilibrium of 73MuH19 was biased to a closed state. Information about the open–close dynamics obtained by HS-AFM correlate well with the *R*_g_ values obtained by SAXS. Therefore, the HS-AFM, as well as SAXS, was a useful tool for selecting compact scFv clones. Based on the evaluation from the mathematical deconvolution of the histogram of the HS-AFM data, an appreciable amount of the open-state existed in the presence of the antigen, while most of the molecules were in the closed-state in 73MuH19, which was supported by the NMR spectra of 73MuH19. These results underscore the importance of producing an scFv whose dynamics are biased to the closed state. 

Finally, we are now proceeding establishment of in vivo live imaging method for AGEs by using engineered scFvs. Open state scFv is assumed to be more susceptible to proteolysis as compared to closed molecule. Our approaches to obtain and evaluate stable as well as compact scFv clones help to accelerate the industrial and therapeutic application of scFvs.

## 4. Materials and Methods

### 4.1. Enzymes and Other Reagents

Restriction and DNA-modifying enzymes were purchased from Takara Bio (Shiga, Japan), New England Biolabs (Ipswich, MA, USA), Promega (Madison, WI, USA), Stratagene (San Diego, CA, USA), Invitrogen (Carlsbad, CA, USA), Toyobo (Osaka, Japan), or Nippon Gene (Tokyo, Japan). Chemicals were obtained from Wako Pure Chemical Industries (Osaka, Japan), Nacalai Tesque (Kyoto, Japan), Sigma (St. Louis, MO, USA), Invitrogen (Waltham, MA, USA), or Lonza (Basel, Switzerland). Amino acid derivatives and resins were purchased from Tokyo Chemical Industry (Tokyo, Japan), Watanabe Chemical Industries (Hiroshima, Japan), or Novabiochem (Billerica, MA, USA). All other reagents were of the highest grade available from commercial sources. 

### 4.2. Preparation of Glycolaldehyde-Modified Bovine Serum Albumin (GA-BSA) and N^α^-(Carbobenzyloxy)-GA-Pyridine (CBZ-GA-Pyridine)

GA-BSA and CBZ-GA-pyridine were prepared using the procedures described previously [21]. In brief, GA-BSA was prepared by incubating BSA (25 mg/mL) with 50 mM glycolaldehyde at 37 °C for 12 h. The reaction mixtures were dialyzed with PBS (137 mM NaCl, 2.7 mM KCl, 10 mM Na_2_HPO_4_, 1.8 mM KH_2_PO_4_) at room temperature. GA-modified *N*^α^-(carbobenzyloxy)-l-lysine (CBZ-Lys) was prepared by incubating 30 mM CBZ-Lys with 900 mM glycolaldehyde at 37 °C for 24 h in PBS. The crude products were purified by reverse-phase high performance liquid chromatography (HPLC) using a COSMOSIL 5C_18_-AR-II column (Nacalai Tesque) with a gradient of water/acetonitrile containing 0.1% trifluoroacetic acid (TFA). The identity of CBZ-GA-pyridine was confirmed by using ^1^H-NMR, ^13^C-NMR, and ESI mass spectrometry analysis. 

### 4.3. Preparation of Biotinylated Peptide Containing GA-Pyridine

Biotinylated peptide (Bio-Gly-Ala-Gly-Lys-Gly-Ala-NH_2_) was synthesized manually using a 9-fluorenylmethoxycarbonyl (Fmoc)-based solid-phase strategy and prepared in the C-terminal amide form, as previously described [24]. The biotinylated peptide containing GA-pyridine (Bio-Gly-Ala-Gly-[GA-pyridine]-Gly-Ala-NH_2_) was prepared by incubating 33 mM biotinylated peptide with 1 M glycolaldehyde at 37 °C for seven days in PBS. The biotinylated peptide containing GA-pyridine was purified by reverse-phase HPLC using a COSMOSIL 5C_18_-AR-II column (Nacalai Tesque) with a gradient of water/acetonitrile containing 0.1% TFA. The purity of the biotinylated peptide containing GA-pyridine was confirmed by analytical reverse-phase HPLC. The identity of the peptide was confirmed by fast atom bombardment mass spectral analysis.

### 4.4. Selection of AGE73scFv by Phage Display

The VH and VL genes of the antibody were cloned into the phagemid vector pCANTAB 5E according to the manufacturer’s manual (GE Healthcare, Little Chalfont, UK) [25] and connected by a (Gly-Gly-Gly-Gly-Ser)_3_ linker. We inserted a FLAG-tag and hexahistidine-tag between the scFv and E-tag. The scFv library was transformed into *E. coli* XL1-Blue electrocompetent cells (Stratagene) and phage particles were obtained by co-infecting with a M13KO7 helper phage. The amplified phages displaying scFv were purified and concentrated by polyethylene glycol (PEG) precipitation, and used for bio-panning. We immobilized GA-BSA (1.5 mL of 20 µL/mL) onto the immunotube (Nunc, Waltham, MA, USA) overnight at 37 °C, performed three washings with PBS, and blocked with PBS containing 2% skim milk for 2 h at 37 °C. The scFv phage library was added to a GA-BSA-coated immunotube, which was incubated for 2 h at 25 °C, then washed three times with PBS-T (PBS containing 0.05% Tween20) in the first round. In each of the second, third, and fourth rounds of panning, the washing was performed 10 times. The bound phages were eluted with 0.1 M glycine-HCl (pH 2.2) containing 1 mg/mL BSA and neutralized with 2 M Tris-HCl (pH 9.1). The eluted phages were amplified by infection into XL1-Blue cells, and the amplified phages were applied to the next-round of panning.

### 4.5. Phage Enzyme-Linked Immunosorbent Assay (ELISA)

We immobilized GA-BSA (100 µL/well of 40 µL/mL) onto a 96-well microplate (Nunc) overnight at 4 °C, washed the microplate three times with PBS-T, and blocked with PBS containing 2% skim milk for 2 h at 37 °C. After washing, scFv display phage clones were added (100 µL/well) to the GA-BSA-coated wells, and incubated for 1 h at 25 °C. The wells were washed three times with PBS-T, and then horseradish peroxidase (HRP)-conjugated anti-M13 antibody was added at a dilution of 1:5000 (100 µL/well) and the wells were incubated for 1 h at 25 °C. The wells were washed three times with PBS-T and then once with PBS. TMB Microwell Peroxidase Substrate solution was added (50 µL/well) and the wells were incubated for an additional 4 min. Finally, 1 M HCl was added to stop the reaction, and the absorbance at a wavelength of 450 nm was measured with a plate reader. 

### 4.6. Expression and Purification of scFv in E. coli

The obtained scFv genes were introduced into pETCF, the modified pET-22b(+) plasmid (Figure S8). The FLAG-tag was inserted into the N-terminal side of a hexahistidine-tag. The scFv genes were introduced by using *Nde* I and *Not* I restriction enzyme sites. 

The expression and purification of scFv were performed as described previously [26,27]. Briefly, when *E. coli* strain BL21(DE3) cells (Novagen, Billerica, MA, USA) containing the expression plasmid reached an OD_600nm_ of 0.6, isopropyl-β-d-1-thiogalactopyranoside (IPTG) was added to a final concentration of 1 mM. The cells were grown at 25 °C for 20 h, then harvested by centrifugation. For the production of isotopically-labeled scFv, Rosetta2 (DE3) (Novagen) was used for expression. The transformed cells were inoculated into 30 mL of TB medium containing ampicillin (100 mg/L), chloramphenicol (30 mg/L), and d-glucose (10 g/L). Rosetta2 (DE3) cells containing the expression plasmid of the scFv were inoculated into LB medium containing ampicillin (100 mg/L), chloramphenicol (30 mg/L) and d-glucose (10 g/L), and cultured overnight at 37 °C. The cultured cells were collected by centrifuge (5000 rpm); resuspended in 300 mL of TB medium containing ampicillin (100 mg/L), chloramphenicol (30 mg/L), and D-glucose (10 g/L); and stirred (180 rpm) at 37 °C until reaching an optical density at 600 nm reached at 2.5–3.0. Then the cells were collected by centrifuge (5000 rpm); washed with 30 mL of PBS; collected again by centrifuge (5000 rpm); and resuspended in 300 mL of M9 medium containing ampicillin (100 mg/L), chloramphenicol (30 mg/L), IPTG (1 mM), d-glucose (u-^13^C) (10 g/L), and ^15^NH_4_Cl (1 g/L); and cultured overnight at 25 °C. We also prepared inversely-labeled samples under a ^15^N background, as described previously [28]. Briefly, 1 g/L of nonlabeled amino acid (A, K, M or R) or a combination of non-labeled amino acids (F/Y/W or I/L/V) was added to the M9 medium. Considering the previous results from inversely amino acid selective-labeling [28,29] and the amino acid biosynthesis pathway of *E. coli* [30], we selected a single amino acid or a combination of amino acids to avoid isotope scrambling.

The scFv was obtained as inclusion bodies, which were solubilized by 6 M guanidine HCl (GdnHCl). The scFvs were refolded on a Ni-NTA Superflow (QIAGEN, Venlo, The Netherlands) column by gradually reducing the concentration of GdnHCl. The refolded scFv was eluted and further purified by gel filtration column chromatography using a Superdex 75 10/300 GL column (GE Healthcare).

### 4.7. Immunohistochemistry

Atherosclerotic lesion specimens of human aortas were embedded in Tissue-Tek OCT (Sakura Finetek, Tokyo, Japan), snap-frozen in liquid nitrogen and stored at −80 °C. The tissues were sectioned and fixed with cold acetone. The sections were treated with the following primary antibody molecules: mouse anti-GA-pyridine monoclonal antibody, 2A2 (Cosmo Bio, Tokyo, Japan) [22,31] or AGE73scFv. The sections treated with AGE73scFv were further incubated with anti-tetra His antibody (QIAGEN) as the secondary antibody. All sections were subsequently treated with an HRP-conjugated anti-mouse IgG antibody (Bio-Rad, Hercules, CA, USA), and visualized with 3,3′-diaminobenzidine.

### 4.8. Surface Plasmon Resonance (SPR)

The affinity of the scFv for GA-pyridine was evaluated by using a Biacore T100 SPR system (GE Healthcare) [32]. The biotinylated peptide containing GA-pyridine (Bio-Gly-Ala-Gly-[GA-pyridine]-Gly-Ala-NH_2_) was immobilized on an SA sensor chip (GE Healthcare) with a running buffer of HBS-EP (10 mM HEPES pH 7.4, 150 mM NaCl, 3 mM EDTA, 0.005% Tween 20). Various concentrations of the scFv solutions were injected into the antigen-immobilized sensor chip under a continuous flow rate of 50 µL/min. The sensorgrams were normalized by subtracting the response from a reference cell on which the biotinylated peptide without GA-pyridine was immobilized.

### 4.9. Construction of the Mutant scFv Library

Random mutagenesis of the VH or VL gene was performed by error-prone PCR [33,34,35]. Briefly, the VH or VL gene was amplified by Gene *Taq* (Nippon Gene) by adding 0.7–1.0 mM MnCl_2_. The PCR program consisted of 30 cycles of denaturation at 98 °C for 30 sec, primer annealing at 55 °C for 1 min, and primer extension at 74 °C for 1 min. The PCR products were purified and cloned into the pCANTAB 5E containing the VH or VL gene of scFv. The scFv mutant library was transformed into *E. coli* XL1-Blue electrocompetent cells (Stratagene), and the phage particles were obtained by co-infection with M13KO7 helper phage. The phage amplification and the bio-panning were performed using methods similar to those described in the previous section with slight modification. In the bio-panning, binding between the phage-displaying mutant scFv library, and GA-BSA was carried out at 37 °C, instead of 25 °C, to obtain stable scFv clones.

### 4.10. Isothermal Titration Calorimetry (ITC)

The thermodynamic parameters of the interactions between scFv and CBZ-GA-pyridine were measured with a MicroCal Auto-iTC200 (Malvern, UK). A 50 µM aliquot of the CBZ-GA-pyridine was titrated with 5 µM of scFv in HBS-EP buffer at 25 °C. The titration curves were analyzed by using MicroCal Origin Software (Malvern, Malvern, UK). The measurement at 37 °C was performed by using Nano ITC (TA Instruments, New Castle, DE, USA). The dissociation constants and thermodynamic parameters were calculated by the one set of sites fitting model. 

### 4.11. Differential Scanning Calorimetry (DSC)

The thermal stability of scFvs was measured with a MicroCal VP-Capillary DSC (Malvern) at a heating rate of 1 °C/min and scFv concentration of 0.5 mg/mL in PBS (137 mM NaCl, 2.7 mM KCl, 10 mM Na_2_HPO_4_, 1.8 mM KH_2_PO_4_). The melting temperatures (*T*_m_) were calculated by the non-two-state model.

### 4.12. Small-Angle X-Ray Scattering (SAXS)

Small-angle X-ray scattering measurements were performed with a Bio-SAXS-1000 system (Rigaku, Tokyo, Japan). Scattering data were taken every 15 min with a total exposure time of 2 h. The *R*_g_ value was estimated by using the ATSAS software package [36] and Guinier approximation [37]. The *R*_g_ value was calculated by the equation

(1) $$\ln\left[I\left(q\right)\right]\;≅\;-\frac{R_{g}^{2}}{3}q^{2}\;+\;\ln[I(0)]$$

### 4.13. High-Speed Atomic Force Microscopy (HS-AFM)

We used a laboratory-built tapping mode HS-AFM apparatus [38,39]. The probe tip was grown on the tip of a cantilever (Olympus, Tokyo, Japan) with electron beam deposition using a scanning electron microscope and was further sharpened by argon plasma etching [40]. Samples were immobilized on the poly-l-lysine-modified mica. A 2 µL aliquot of poly-l-lysine (1.0 μg/mL) was deposited on the surface of the mica for 3 min. After washing with 200 µL of PBS, scFv diluted in PBS at 7.5 ng/mL or 7.5 µg/mL was deposited on the surface of poly-l-lysine-modified mica. The complex of scFv with CBZ-GA-pyridine was prepared by incubating scFv and CBZ-GA-pyridine, and then the free CBZ-GA-pyridine was removed by ultrafiltration with a molecular weight cutoff of 10 kDa before the immobilization on the mica surface. The surface of the mica was washed with 20 µL of PBS, and the cantilever was soaked in PBS or PBS containing CBZ-GA-pyridine, respectively, for observation of the free or complex states. AFM observations were performed at room temperature by using a scan area of 50 × 50 nm^2^ with 80 × 80 pixels and a scan rate of 10 frames/s. A 3 × 3 pixel-average filter was used to reduce noise. The distances between the VH and VL domain in the open and closed state were calculated from the histograms fitted by a Gaussian distribution using Origin software.

### 4.14. NMR Spectroscopy

For all NMR measurements, the sample solution consisted of 50 mM phosphate buffer (pH direct read of 6.5) containing 150 mM NaCl in 90% H_2_O/10% ^2^H_2_O with a protein concentration of 0.3 mM. Data were processed using the NMRpipe program package [41]. All multidimensional NMR experiments were performed on Inova 800, 600, or 500 MHz NMR spectrometers (Agilent, Santa Clara, CA, USA) equipped with a room temperature probe.

## Figures and Tables

**Table 1:** Characterization of scFv clones.

scFv Clones	^1^ *K*_D_ × 10^−8^ (M)		^2^ *T*_m_ (°C)	^3^ *R*_g_ (Å)
	25 °C	37 °C		
AGE73scFv	13.4 ± 0.5	68.4 ± 13.2	62.8 ± 0.02	25.1 ± 0.5
73MuL-V94A	5.3 ± 1.3	14.9 ± 2.7	62.7 ± 0.02	22.5 ± 0.6
73MuH19	4.7 ± 1.1	23.0 ± 5.3	65.0 ± 0.02	20.5 ± 0.8

^1^ Obtained by ITC measurements; ^2^ Obtained by DSC measurements; ^3^ Obtained by SAXS measurements.

## Supplementary Materials

Supplementary Materials are available online.
