# Supplementary material for: Production of Single-Chain Fv Antibodies Specific for GA-Pyridine, an Advanced Glycation End-Product (AGE), with Reduced Inter-Domain Motion
Source: Molecules. 2017 Oct 10;22(10):1695. doi: 10.3390/molecules22101695 (PMC6151396; doi:10.3390/molecules22101695)
Supplement: Supplementary file 1 [file molecules-22-01695-s001.pdf]

**Supplementary Materials**

**Table S1.** Retention volume of scFv clones in the gel filtration chromatography.

| scFv clones | Retention volume (mL) |
|---|---|
| AGE73scFv | 12.8 |
| 73MuL-S30P | 12.8 |
| 73MuL-S56P | 13.0 |
| 73MuL-V94A | 14.4 |

**Table S2.** Mutated residues in the selected clones by bio-panning from the second and third library.

| Second library | | | |
|---|---|---|---|
| Mutations in VL | Number of clone | Mutations in VL | Number of clone |
| V94A | 5 | S56P | 1 |
| K39R | 2 | V58A | 1 |
| M78V | 2 | S65G | 1 |
| T22A | 1 | Y71N | 1 |
| C23R | 1 | E81G | 1 |
| S27L | 1 | D82G | 1 |
| S27aG | 1 | A84G | 1 |
| V28R | 1 | S91G | 1 |
| S30P | 1 | T97S | 1 |
| S43P | 1 | G100E | 1 |
| S52Y | 1 | | |

| Third library | |
|---|---|
| Mutations in VH | Number of clone |
| D100hG | 4 |
| F40L | 1 |
| Y47H | 1 |
| L53F | 1 |
| D100hV | 1 |
| D100hG | 4 |

**Table S3.** The population of open and closed states of scFvs.

| scFv clones | Distance between two peaks (nm) | Percentage | State |
|---|---|---|---|
| AGE73scFv | 2.8 ± 0.3 | 7.0 | Closed |
| | 4.7 ± 0.7 | 93.0 | Open |
| AGE73scFv + CBZ-GA-pyridine | 2.8 ± 0.4 | 59.1 | Closed |
| | 4.2 ± 0.5 | 40.9 | Open |
| 73MuL-V94A | 2.7 ± 0.5 | 65.0 | Closed |
| | 4.3 ± 0.5 | 35.0 | Open |
| 73MuL-V94A + CBZ-GA-pyridine | 2.6 ± 0.4 | 63.8 | Closed |
| | 4.1 ± 0.5 | 36.2 | Open |
| 73MuH19 | 2.4 ± 0.7 | 81.3 | Closed |
| | 4.4 ± 0.5 | 18.7 | Open |
| 73MuH19 + CBZ-GA-pyridine | 1.9 ± 0.3 | 39.3 | Closed |
| | 2.8 ± 0.9 | 60.7 | Closed |

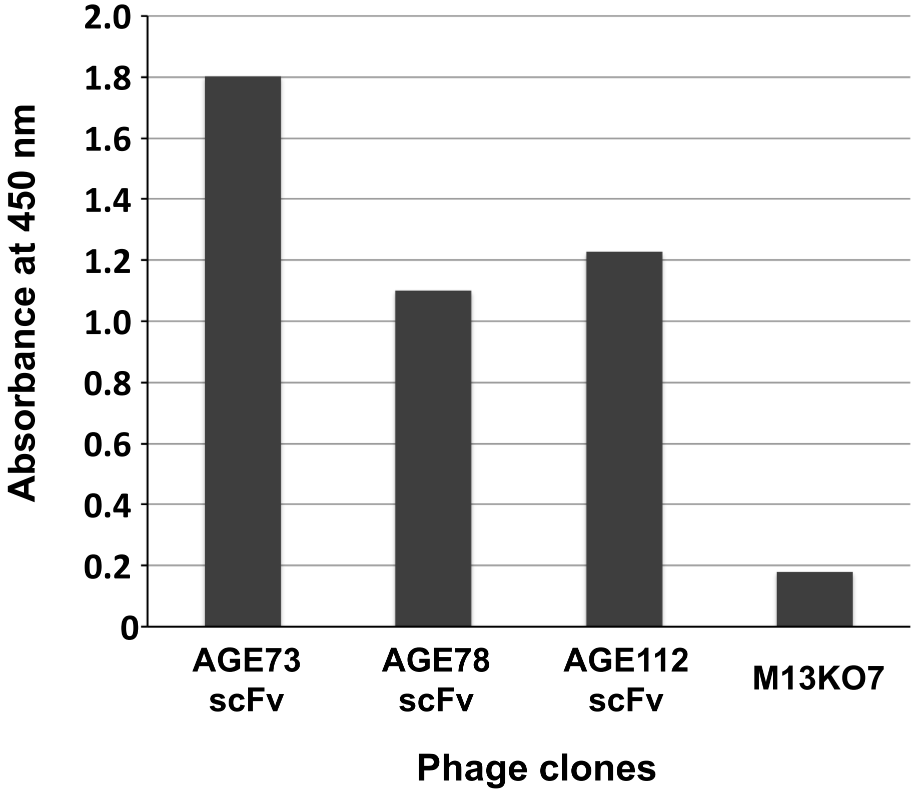


**Figure S1.** Binding of scFv-display phage clones from GA-KLH immunized mouse antibody library in phage ELISA to GA-BSA. M13KO7 helper phage was used as a control sample.

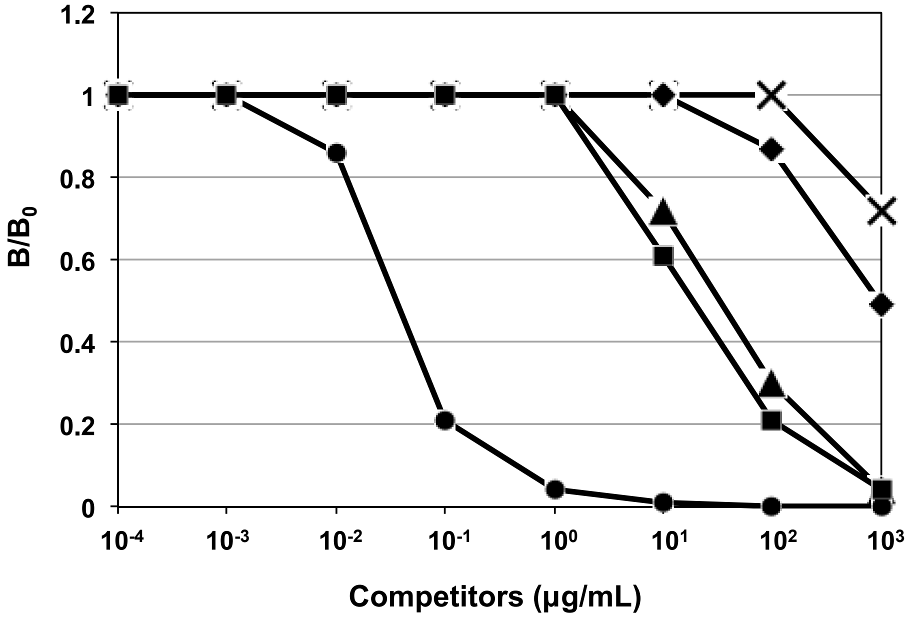


**Figure S2.** Immunoreactivity of AGE73scFv. GCA-BSA, Glc-BSA, MGO-BSA and GO-BSA were prepared in the similar way as described previously [21]. Competitive ELISAs were performed using the similar procedures described previously [21]. Reactivity of AGE73scFv to GA-BSA was analyzed using several AGE-modified BSAs as competitors. AGE-modified BSAs utilized are as follows: GA-BSA (circle), GCA-BSA (square), Glc-BSA (triangle), MGO-BSA (diamond) and GO-BSA (cross).

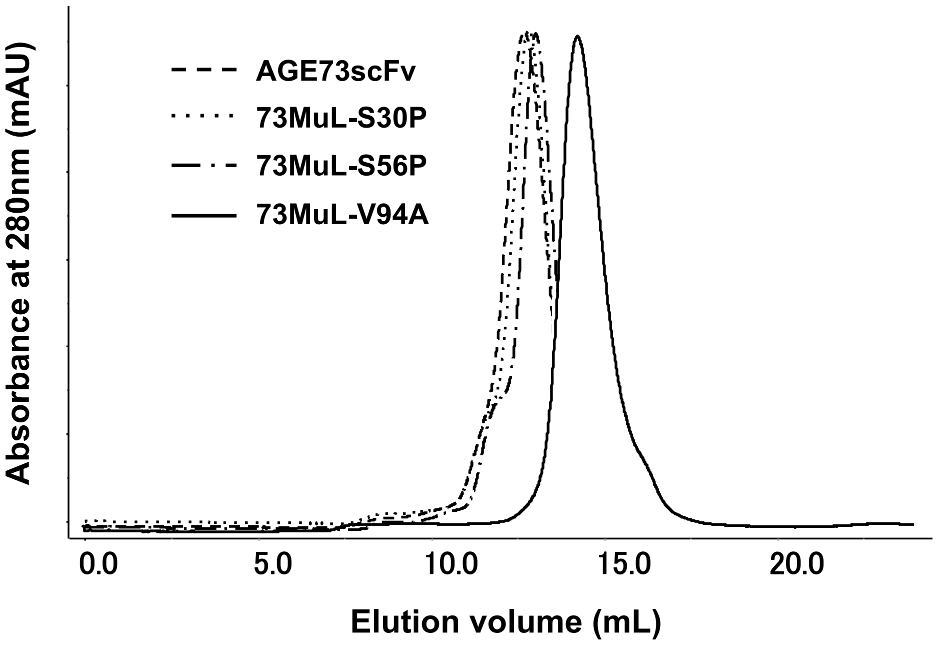


**Figure S3.** Gel filtration chromatography. The lines represent AGE73scFv (dashed line), 73MuL-S30P (dotted line), 73MuL-S56P (dashed and dotted line) and 73MuL-V94A (solid line), respectively.

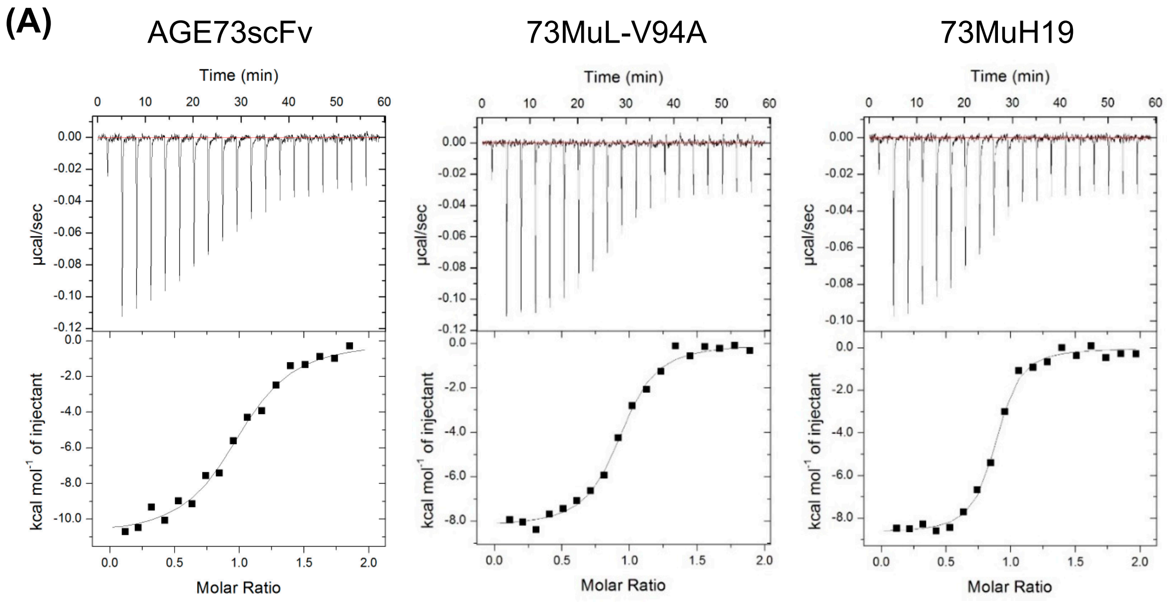

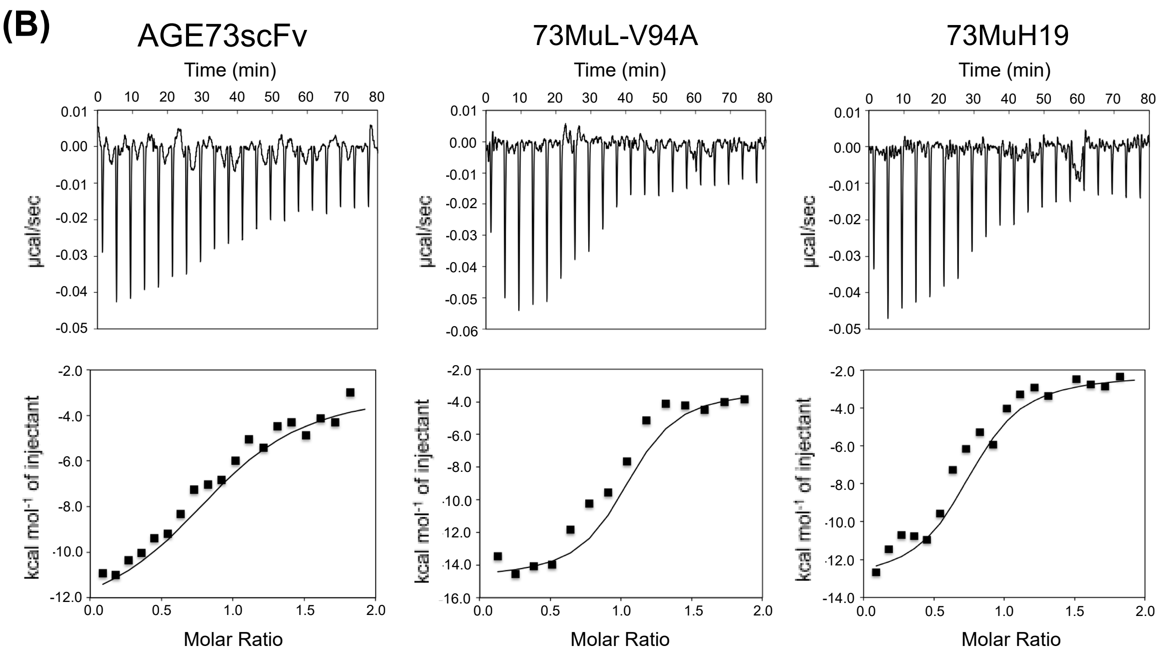


**Figure S4.** Titration calorimetry of the interactions between scFv and CBZ-GA-pyridine at 25 °C (A) and 37 °C (B). Top, typical calorimetric titration of scFv (5 µM) with CBZ-GA-pyridine (50 µM) in HBS-EP buffer; bottom, interaction plot of the data calculated from the raw data. The solid line corresponds to the best fitting curve using the one set of sites fitting model.

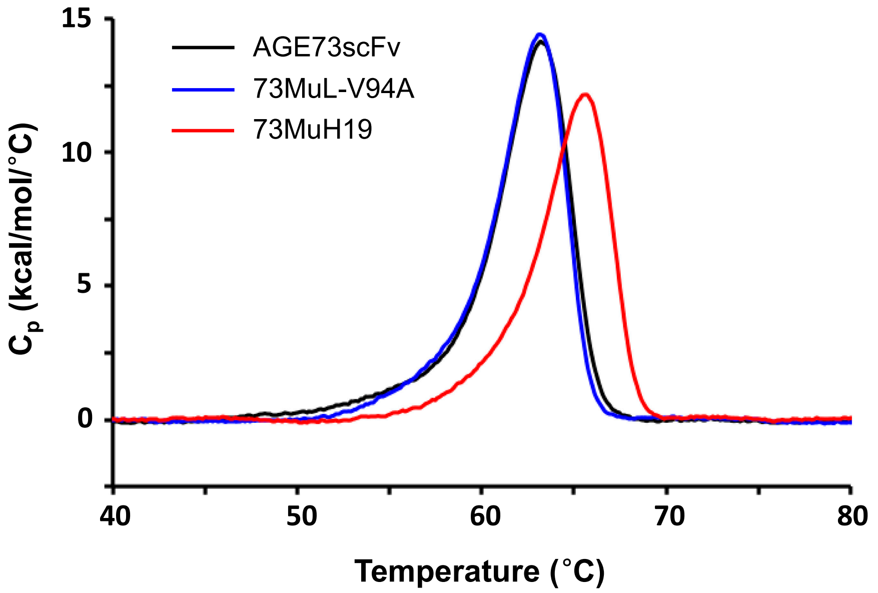


**Figure S5.** DSC thermogram showing $T_m$ values of AGE73scFv (black), 73MuL-V94A (blue), and 73MuH19 (red). The scFv concentration was 0.5 mg/ml in PBS. The melting temperatures ($T_m$) were calculated by the non-two-state model.

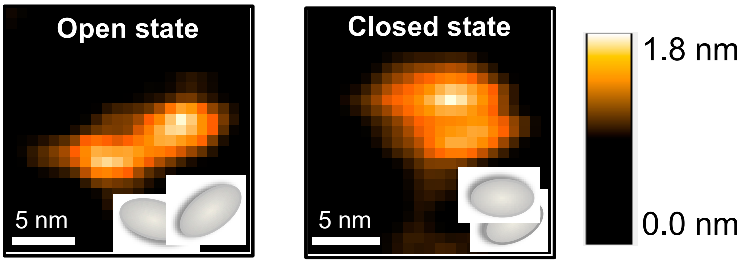


**Figure S6.** Representative snap shots of HS-AFM in the open (left panel) and closed (right panel) states of the scFv.

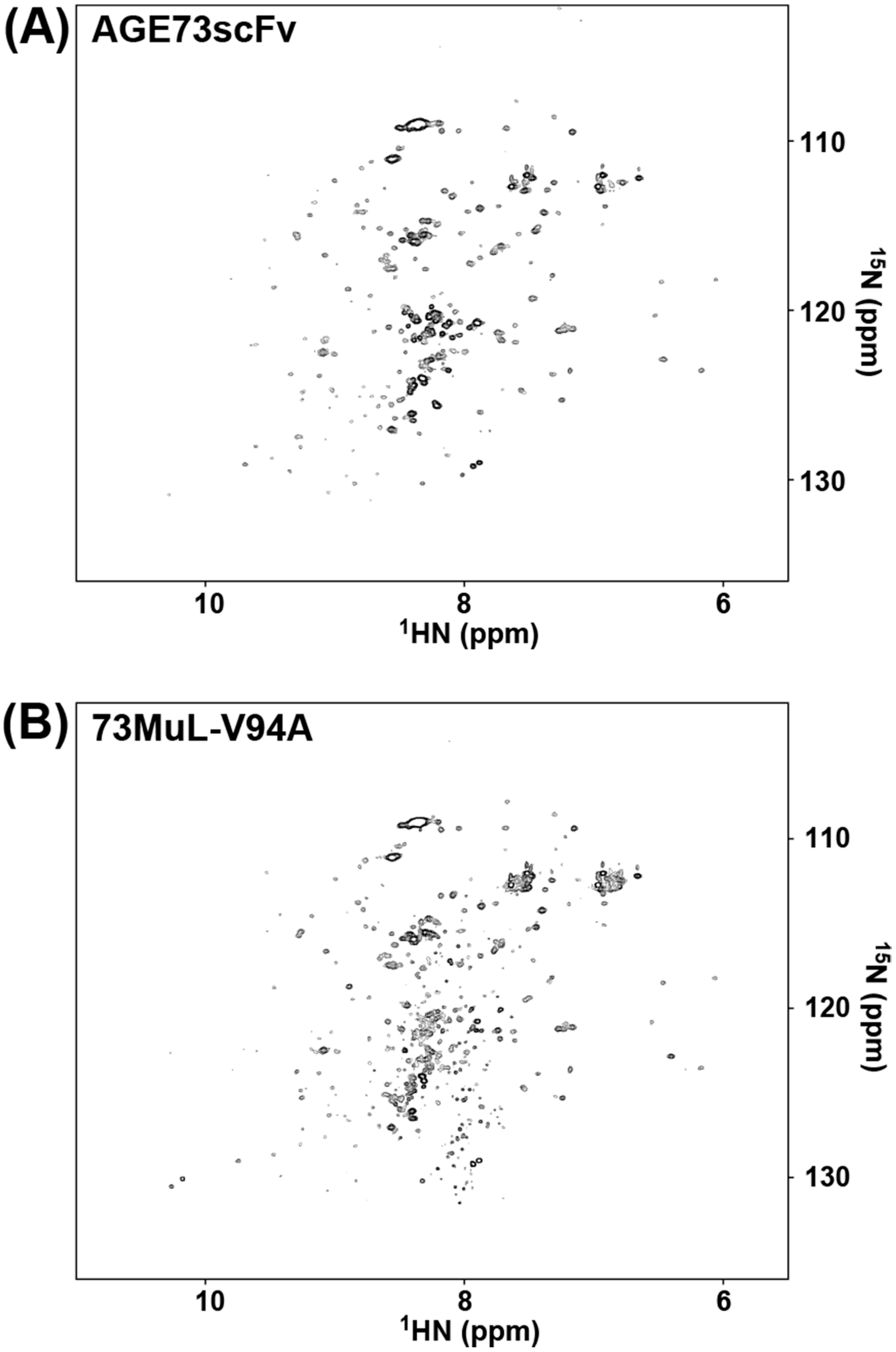

**Figure S7.** The $^1H$-$^{15}N$ HSQC spectra of AGE73scFv (A) and 73MuL-V94A (B) in the antigen free state at 37 °C.

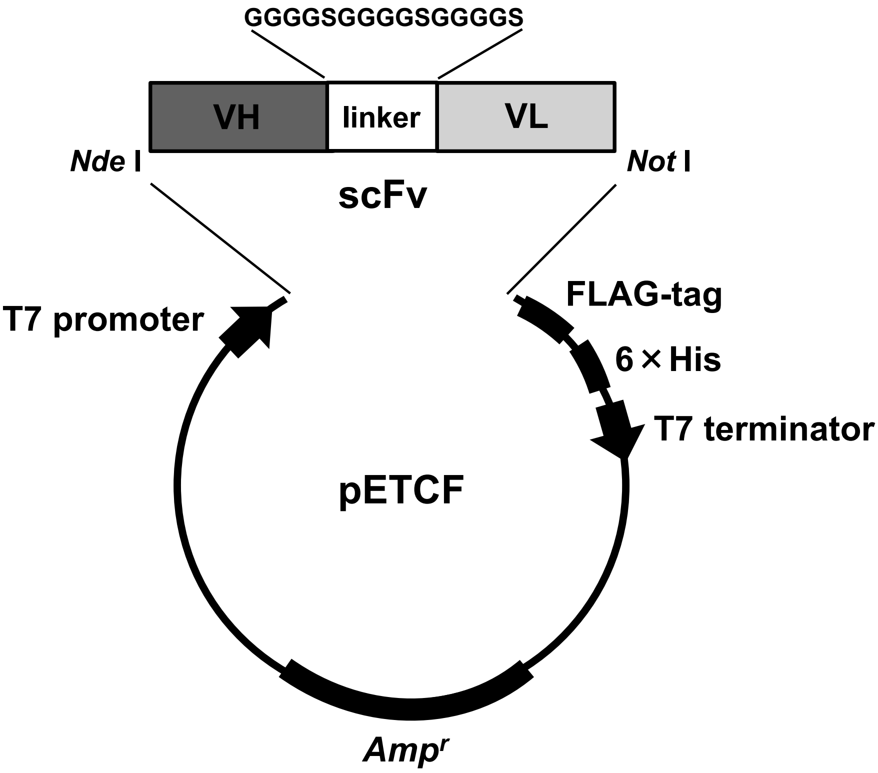


**Figure S8.** Expression vector of the single-chain Fv antibodies specific for GA-pyridine.
